# Zusammenhang von Belastungen der Lehrpersonen und Eltern mit reaktiver und proaktiver Aggression der Kinder: Bedeutung von Emotionsregulation und kooperativem Verhalten

**DOI:** 10.1007/s42278-022-00149-8

**Published:** 2022-05-30

**Authors:** Markus P. Neuenschwander, Ilona Rösti, Vanessa Prieth, Janine Bölsterli, Alafia Zavery

**Affiliations:** Zentrum Lernen und Sozialisation, Pädagogische Hochschule der Fachhochschule Nordwestschweiz, Bahnhofstrasse 6, 5210 Windisch, Schweiz

**Keywords:** Reaktive und proaktive Aggression, Emotionsregulation, Kooperatives Verhalten, Familie, Schule, Reactive and proactive aggression, Emotion regulation, Cooperative behavior, Family, School

## Abstract

Emotionsregulation und kooperatives Verhalten in belastenden Situationen spielen eine zentrale Rolle bei der Erklärung von proaktiver und reaktiver Aggression der Kinder. Wie sehr erklären die Emotionsregulation und das kooperative Verhalten den Effekt von Belastungen der Eltern bzw. Lehrpersonen auf die reaktive und proaktive Aggression der Kinder? Ausgewertet wurden querschnittliche Fragebogendaten von Eltern und Lehrpersonen, die *N* = 158 Kinder mit Verhaltensauffälligkeiten in Kindergarten und Primarstufe einschätzten. Strukturgleichungsmodelle zeigen, dass die Belastung der Eltern bzw. Lehrpersonen mit der Emotionsregulation und dem kooperativen Verhalten indirekt mit der reaktiven und proaktiven Aggression zusammenhängt. Die Ergebnisse verdeutlichen die Bedeutsamkeit einer frühzeitigen Förderung der Emotionsregulation und des kooperativen Verhaltens.

## Abstract (en)

Emotion regulation and cooperative behavior in stressful situations contribute to the explanation of children’s reactive and proactive aggression. How strongly do emotional regulation and cooperative behavior explain the effect of teachers’ and parents’ stress on children’s reactive and proactive aggression? Cross sectional questionnaire data from teachers and parents assessing *N* = 158 children with behavioral problems in kindergarten and primary school were analyzed. Structural equation models show that teacher’s or parent’s stress is related to emotion regulation and cooperation, indirectly to reactive and proactive aggression. The results highlight the importance of early support of emotion regulation and cooperative behavior.

## Einleitung

Aggressives Verhalten der Kinder im Kindergarten und in der Primarstufe beeinträchtigt nicht nur das Wohlbefinden der Mitschüler*innen, sondern wirkt sich auch negativ auf das Lernen und die Schullaufbahn der betroffenen Kinder selbst aus (Vitaro et al. 2005). In der Literatur wird zwischen der reaktiven und der proaktiven Form des aggressiven Verhaltens unterschieden (Dodge et al. 1997; Merk et al. 2005). Während reaktive Aggression eher auf Wahrnehmungs- und Interpretationsprobleme der sozialen Umwelt zurückzuführen ist, ist proaktive Aggression mit problematischen, instrumentellen Strategien der Zielerreichung in sozialen Situationen verbunden (Lohbeck et al. 2014). Dysfunktionale Informationsverarbeitungsprozesse spielen beim Entstehen von reaktiver und proaktiver Aggression eine zentrale Rolle (Crick und Dodge 1994). Entsprechend zeigte frühere Forschung Zusammenhänge zwischen der Emotionsregulation und kooperativem Verhalten von Kindern mit proaktiver und reaktiver Aggression (Lohbeck et al. 2014).

Allerdings wurden die aggressiven Verhaltensformen insbesondere in der Selbstwahrnehmung der Kinder erfasst (Skripkauskaite et al. 2015). Seltener wurden sie in der Fremdwahrnehmung aus verschiedenen Perspektiven (Lehrpersonen, Eltern) und in verschiedenen Kontexten (z. B. Schule und Familie) vergleichend untersucht (Gratz und Roemer 2004; Youngstrom et al. 2000). Insbesondere in der Kindheit können Selbstwahrnehmungen mit Validitätsproblemen konfrontiert sein. Eine kontext-vergleichende Sicht liefert Hinweise, ob sich der Einfluss der Determinanten der proaktiven und reaktiven Aggression wie beispielsweise die Belastungen der Lehrpersonen (Schule) und Eltern (Familie) zwischen den Kontexten unterscheiden (Brendgen et al. 2001).

Viele Studien zeigten Effekte von aggressivem Verhalten im Unterricht auf die Belastungen der Lehrpersonen und Eltern (Amstad und Müller 2020; Podolski und Nigg 2001; Siekkinen et al. 2013). Jedoch wurde der Effekt von Belastungen der Bezugspersonen (Lehrpersonen bzw. Eltern) aufgrund von Verhaltensauffälligkeiten der Kinder auf die reaktive und proaktive Aggression der Kinder seltener bearbeitet (Bottiani et al. 2019; Siekkinen et al. 2013). Es wird ein Kreisprozess vermutet, wonach Verhaltensauffälligkeiten von Kindern (dazu gehören auch die reaktive und proaktive Aggression) die Belastungen der Lehrpersonen bzw. Eltern erhöhen, welche ihrerseits die reaktive und proaktive Aggression der Kinder begünstigen (Bottiani et al. 2019). Belastungen von Lehrpersonen bzw. Eltern können die Informationsverarbeitungsprozesse der Kinder beeinträchtigen und dadurch aggressives Verhalten begünstigen (Familie: Podolski und Nigg 2001; Schule: Jeon et al. 2019). Während viele Studien reaktiv und proaktiv aggressives Verhalten von Jugendlichen untersuchten, wurde es in der Kindheit seltener analysiert (Olson et al. 2011). Es fehlen gesicherte Erkenntnisse, wie Belastungen, Emotionsregulation und kooperatives Verhalten mit reaktiver und proaktiver Aggression in den Kontexten Schule und Familie zusammenhängen (Forschungslücke). Erkenntnisse zur Bedeutung der Emotionsregulation und des kooperativen Verhaltens ermöglichen, Interventionen zur Förderung der Emotionsregulation und des kooperativen Verhaltens zu entwickeln, die zwischen dem schulischen und familiären Kontext koordiniert sind. Damit kann ungünstigen Verläufen der sozio-emotionalen Entwicklung von Kindern vorgebeugt werden. Eine abgestimmte Förderung der sozio-emotionalen Entwicklung in Schule und Familie könnte die reaktive und proaktive Aggression von Kindern reduzieren. Befunde zur Behebung der Forschungslücke erlauben, ein empirisch basiertes, kontextübergreifendes Interventionsprogramm zu entwickeln (Neuenschwander et al. 2022).

Im vorliegenden Artikel werden daher anhand einer Stichprobe von Kindern mit Verhaltensauffälligkeiten die Fragen bearbeitet, wie die Belastungen von Lehrpersonen bzw. Eltern aufgrund von Verhaltensauffälligkeiten der Kinder mit kooperativem Verhalten bzw. Emotionsregulation sowie reaktiver und proaktiver Aggression von Kindern im Kindergarten sowie in der Primarstufe zusammenhängen und wie übereinstimmend diesbezüglich die Lehrpersonen- und die Elterneinschätzung ausfallen.

### Aggressives Verhalten und Informationsverarbeitung

Aktuell werden mehrere Modelle der Emotionsverarbeitung diskutiert. Pekrun (2006) beschrieb ein Modell der Emotionsregulation im Leistungskontext. Crick und Dodge (1994) formulierten ein häufig verwendetes Modell der Informationsverarbeitung zur Erklärung von aggressivem Verhalten in sozialen Situationen, das eine gute Basis zur Bearbeitung der Forschungsfrage bildet. Sie gehen von einem Kreisprozess aus, in welchem Informationen in Abstimmung mit früheren Erfahrungen und Handlungskonsequenzen, erlernten Regeln und sozialen Kompetenzen schrittweise verarbeitet werden. Sozial-kognitive Kompetenzen des Kindes beeinflussen diese Informationsverarbeitungsprozesse. Kinder, die mit anderen Kindern kooperieren können, verfügen über soziale Strategien zur Erreichung ihrer Ziele, die sozial akzeptiert sind (Kanning 2009). Entsprechend belegte frühere Forschung den engen negativen Zusammenhang von kooperativem Verhalten mit reaktiver und proaktiver Aggression (Lohbeck et al. 2014). Wir vermuten daher, dass kooperatives Verhalten mit einer geringeren Neigung zu reaktiver und proaktiver Aggression einhergeht.

Gemäß Crick und Dodge (1994) beeinflusst die Interpretation von sozialen Umweltreizen die Emotionsregulation. Kinder, die soziale Umweltreize fälschlicherweise für sich als bedrohlich wahrnehmen, reagieren darauf eher mit heftigen negativen Emotionen wie Wut oder Angst. In Konsequenz ist ihre Neigung zu aggressivem Verhalten erhöht. Kinder, die die eigenen Emotionen steuern können, reagieren weniger impulsiv und weniger aggressiv auf Herausforderungen aus ihrer Umwelt (White et al. 2012). Verschiedene Studien konnten einen Zusammenhang zwischen der Emotionsregulation und der Neigung zu aggressivem Verhalten nachweisen (DeWall et al. 2011; Röll et al. 2012; Skripkauskaite et al. 2015). Hubbard et al. (2010) berichteten Effekte der Emotionsregulation sowohl auf reaktive als auch auf proaktive Aggression. Entsprechend nehmen wir einen positiven Zusammenhang zwischen einer geringen Emotionsregulation und der Ausprägung von reaktiver und proaktiver Aggression an.

### Aggressives Verhalten und Belastungen der Lehrpersonen und Eltern

Sowohl soziale Informationen von Gleichaltrigen als auch von Lehrpersonen und Eltern beeinflussen die Informationsverarbeitung von Kindergarten- und Primarschulkindern. Belastungen der Lehrpersonen bzw. Eltern aufgrund von Verhaltensauffälligkeiten der Kinder können mit negativem Stress einhergehen und sich gegenüber den Kindern in ungünstigen Verhaltensweisen äußern, die von den Kindern als weniger sensitiv und fürsorglich wahrgenommen werden (Bottiani et al. 2019; Brendgen et al. 2001; Jennings und Greenberg 2009; Koenen et al. 2019; Sidor et al. 2018). Unkooperatives oder unkontrolliertes Verhalten von Lehrpersonen bzw. Eltern kann die proaktive und reaktive Aggression begünstigen (Skriphauskaite et al. 2015). Entsprechend wurden Zusammenhänge zwischen Belastungen der Lehrpersonen bzw. Eltern und reaktiver sowie proaktiver Aggression der Kinder gefunden (Benini et al. 2017; Skriphauskaite et al. 2015).

Ausgehend von Crick und Dodge (1994) wird vermutet, dass der Zusammenhang zwischen den Belastungen von Lehrpersonen bzw. Eltern und der reaktiven und proaktiven Aggression durch die Emotionsregulation der Kinder erklärt wird. Verhaltensweisen von belasteten Lehrpersonen und Eltern können manche Kinder nicht angemessen interpretieren. Solche Fehlinterpretationen tragen dazu bei, dass sie ihre Emotionen weniger regulieren können. Daher hängen ausgeprägte Belastungen der Lehrpersonen bzw. Eltern mit geringer Emotionsregulation zusammen. In Konsequenz zeigen die Kinder aus der Perspektive der Lehrpersonen bzw. Eltern eher reaktiv und proaktiv aggressives Verhalten. Studien zeigten, dass die Emotionsregulation des Kindes den Effekt von belastetem Verhalten der Lehrpersonen bzw. Eltern auf Aggression erklärt (Heidgerken et al. 2004; Hubbard et al. 2010; McCoy und Raver 2011).

Crick und Dodge (1994) postulierten, dass der Effekt von Belastungen der Lehrpersonen bzw. Eltern auf die proaktive und reaktive Aggression der Kinder überdies durch geringes kooperatives Verhalten der Kinder erklärt werden kann. Kinder mit geringen kooperativen Kompetenzen können Fehlinterpretationen des Verhaltens von belasteten Lehrpersonen bzw. Eltern weniger kompensieren, weil sie über weniger Alternativen an kooperativen Verhaltensweisen verfügen (Lohbeck et al. 2014). Wir nehmen daher an, dass ausgeprägte Belastungen von Lehrpersonen bzw. Eltern mit weniger ausgeprägtem kooperativem Verhalten von Kindern zusammenhängen und dass Effekte von belasteten Lehrpersonen bzw. Eltern auf die reaktive und proaktive Aggression durch Emotionsregulation und kooperatives Verhalten der Kinder mediiert werden.

### Vergleich zwischen Schule und Familie

Auch wenn das Modell von Crick und Dodge (1994) Kontexteffekte einschließt, handelt es sich um ein allgemeines, bereichsunspezifisches Modell der Informationsverarbeitung. Entsprechend dürften sich individuelle Verhaltenstendenzen in beiden Kontexten Schule und Familie im interindividuellen Vergleich analog zeigen. Wir nehmen in Weiterführung zu früheren Studien an, dass die Einschätzungen zum aggressiven Verhalten der Kinder zwischen den Lehrpersonen und Eltern korrelieren (Achenbach et al. 1987; Heilig und Pauen 2013; Hintermaier et al. 2019; Youngstrom et al. 2000). Allerdings zeigten Studien, dass Lehrpersonen externalisierende Verhaltensweisen (beispielsweise reaktive und proaktive Aggression) sensitiver einschätzen, während Eltern sensitiver auf internalisierende Verhaltensweise (beispielsweise depressives Verhalten) reagieren (Abikoff et al. 1993; Hintermaier et al. 2019). Es wird vermutet, dass Lehrpersonen die reaktive und proaktive Aggression der Kinder im Durchschnitt höher einschätzen als Eltern. Daher sollen die Konzepte Belastungen der Eltern bzw. Lehrpersonen, Emotionsregulation und Kooperation von Kindern sowie reaktive und proaktive Aggression von Kindern aus den Perspektiven der Lehrpersonen sowie der Eltern erfasst und getrennt aus den beiden Perspektiven analysiert werden.

### Hypothesen

Zusammenfassend werden die folgenden Hypothesen überprüft:

a) Die Ausprägungen von reaktiver und proaktiver Aggression der Kinder korrelieren positiv mit den Einschätzungen von Lehrpersonen und Eltern. b) Die Lehrpersonen schätzen die reaktive und proaktive Aggression der Kinder höher ein als die Eltern.
Emotionsregulation und kooperatives Verhalten von Kindern haben einen negativen Effekt auf proaktive und reaktive Aggression, jeweils erfasst aus den Perspektiven der Lehrpersonen und der Eltern.
Die Ausprägung der Belastungen von Lehrpersonen bzw. Eltern durch die Verhaltensauffälligkeiten der Kinder hat einen negativen Effekt auf das kooperative Verhalten und die Emotionsregulation, erfasst aus den Perspektiven der Lehrpersonen und Eltern.
Die Belastungen der Lehrpersonen bzw. Eltern haben einen positiven Effekt auf die reaktive und proaktive Aggression der Kinder, erfasst aus den Perspektiven Lehrpersonen und Eltern. Diese Effekte werden durch kooperatives Verhalten und Emotionsregulation, aus den Perspektiven der Lehrpersonen und Eltern, mediiert.
Die formulierten Hypothesen gelten gleicherweise im schulischen und familiären Kontext.

## Methode

### Datengrundlage

Die Bearbeitung der Fragestellungen erfolgte anhand von Daten des Projektes Förderung der Selbstregulation in Schule und Familie – FOSSA. In diesem Projekt wurden schulische und familiäre Bedingungen von Selbstregulation und aggressivem Verhalten von Kindern mit Verhaltensauffälligkeiten untersucht sowie überprüft, wie diese modifiziert werden können. Dafür wurden Daten zu Kindern vor und nach einer Intervention sowie in einer Kontrollgruppe erfasst. Schulen (Kindergärten und Primarschulen) der Kantone Aargau, Basel-Landschaft, Bern, Luzern, Solothurn, St. Gallen und Zürich wurden geordnet aufgelistet, zufällig ausgewählt und angefragt. Interessierte Lehrpersonen dieser Schulen füllten bei einer Zusage ein Screening für die ganze Klasse aus und schätzten die Verhaltensweisen sowie familiären Belastungen aller Kinder der Klasse ein, wobei pro Kind in der Regel Aussagen von mehreren unterrichtenden Lehrpersonen bzw. Sonderpädagog*innen vorlagen, um Effekte diskriminierender Etikettierungen zu reduzieren. Auf dieser Basis wurden Kinder mit Verhaltensauffälligkeiten und familiären Belastungen identifiziert und ihre Eltern zur Teilnahme an der Studie angefragt. Bei einer Zusage gaben die Eltern schriftlich ihr Einverständnis. Die Lehrpersonen füllten online einen standardisierten Fragebogen zu den Kindern zu zwei Messzeitpunkten aus, die Eltern erhielten ihn jeweils im selben Zeitraum in Papierform und konnten bei Bedarf eine Übersetzungshilfe anfordern. Alle Daten wurden pseudonymisiert erfasst, Kontaktangaben wurden separat von den Erhebungsdaten aufbewahrt, der Datenschutz wurde eingehalten. Weil im Schuljahr 2019/2020 (Kohorte 1) nicht ausreichend viele Kinder in die Studie einbezogen werden konnten, wurde im Schuljahr 2020/2021 die Stichprobe (Kohorte 2) vergrößert, um aussagekräftige Auswertungen durchführen zu können. Die erste Kohorte startete im Sommer 2019, die zweite Kohorte im Sommer 2020. Für die vorliegenden Analysen wurden die Daten des ersten Messzeitpunkts beider Kohorten ausgewertet. Da bei manchen Variablen die Normalitätsvoraussetzung nicht gegeben war, wurde anhand des *Mann-Whitney-U-Tests *für unabhängige Stichproben überprüft, ob sich die Daten der Kohorte 1 und 2 unterscheiden. Die Ergebnisse zeigten, dass es bei den verwendeten Konzepten des vorliegenden Beitrags keine signifikanten Unterschiede zwischen den beiden Kohorten gab (Eltern: reaktive Aggression *U* = −0,37, *p* = 0,715, *N* = 146; proaktive Aggression: *U* = 0,43, *p* = 0,669, *N* = 145; Emotionsregulation *U* = −0,18, *p* = 0,860, *N* = 147; kooperatives Verhalten *U* = 1,36, *p* = 0,173, *N* = 147; Belastungen *U* = −0,41, *p* = 0,683, *N* = 144; Lehrpersonen: reaktive Aggression *U* = −1,41, *p* = 0,158, *N* = 152; proaktive Aggression: *U* = −1,47, *p* = 0,141, *N* = 152; Emotionsregulation *U* = −1,11, *p* = 0,268, *N* = 153; kooperatives Verhalten *U* = 1,11, *p* = 0,266, *N* = 152; Belastungen *U* = −0,43, *p* = 0,664, *N* = 152). Obwohl die Datenerhebung im Sommer 2020 während der COVID 19-Pandemie durchgeführt wurde, war die Situation an den Schweizer Schulen ähnlich wie die Situation im Vorjahr, so dass keine Effekte der Pandemie auf die untersuchten Konstrukte gefunden werden konnten.

Die Stichprobe umfasst insgesamt *N* = 158 Kinder aus zwei Kohorten. Davon waren 108 Kinder in der Primarstufe (1. bis 3. Klasse) und 50 Kinder im Kindergarten. Im Durchschnitt waren die Kinder 7,1 Jahre alt (Standardabweichung *SD* *=* 1,2) mit einer Spannweite von 5–10 Jahren. Insgesamt nahmen 31 Mädchen und 127 Jungen teil. Die Kinder verteilten sich auf 87 Schulklassen aus 69 Schulen, an denen pro Klasse jeweils 1–4 Kinder teilnahmen und 1–3 Lehrpersonen unterrichteten. Insgesamt sind Daten von 128 Lehrpersonen vorhanden. Die Lehrpersonen waren im Durchschnitt 41,3 Jahre alt (*SD* *=* 11,6) und hatten eine durchschnittliche Berufserfahrung von 14 Jahren (*SD* *=* 10,2). Die Bezugsperson des Kindes (z. B. Eltern), die den Fragebogen ausgefüllt hat, war im Durchschnitt 38,1 Jahre alt (*SD* *=* 5,3). In 130 Fällen handelte es sich dabei um die Mutter, in 17 Fällen um den Vater, bei 11 Fällen fehlten die Angaben.

### Messinstrumente

Die Aggression der Kinder wurde aus Lehrpersonen- sowie Elternperspektive mit den Subskalen reaktive und proaktive Aggression (Dodge und Coie 1987) operationalisiert, welche aus dem Englischen übersetzt wurden. Die *reaktive Aggression* (Beispielitem: Wenn das Kind geärgert oder bedroht wird, wird es schnell wütend und schlägt zu. Lehrpersonen: α = 0,78, *M* = 4,3, *SD* = 1,0, *N* = 152; Eltern: α = 0,78, *M* = 3,2, *SD* = 1,2, *N* = 146) sowie die *proaktive Aggression* (Beispielitem: Das Kind bringt andere Kinder dazu, sich gegen ein Kind zu verbünden, das es nicht mag. Lehrpersonen: α = 0,82, *M* = 3,0, *SD* = 1,1, *N* = 152; Eltern: α = 0,81, *M* = 1,9, *SD* = 0,9, *N* = 144) umfassten je drei Items. Die Konzepte wurden auf einer Skala von 1 „trifft überhaupt nicht zu“ bis 6 „trifft voll und ganz zu“ eingeschätzt.

Für die *Emotionsregulation* wurden vier Items eingeschätzt, welche vom Eltern-Belastungs-Inventar (EBI) nach Tröster (2010) entnommen wurden (Beispielitem: Das Kind reagiert oft sehr heftig, wenn etwas passiert, das es nicht mag. Lehrpersonen: α = 0,84, *M* = 3,6, *SD* = 1,1, *N* = 153; Eltern: α = 0,82, *M* = 3,3, *SD* *=* 1,1, *N* = 146). Für die Emotionsregulation wurde eine Skala von 1 „trifft überhaupt nicht zu“ bis 6 „trifft voll und ganz zu“ verwendet, wobei ein hoher Wert eine geringe Emotionsregulation anzeigt. Das *kooperative Verhalten* umfasste vier Items aus der Lehrereinschätzliste für Sozial- und Lernverhalten nach Petermann und Petermann (2013; Beispielitem: Das Kind arbeitet mit anderen in einer Gruppe zusammen. Lehrpersonen: α = 0,84, *M* = 2,9, *SD* = 0,5, *N* = 151; Eltern: α = 0,65, *M* = 3,3, *SD* = 0,4, *N* = 136). Für die Einschätzung des kooperativen Verhaltens wurde eine Skala von 1 „Verhalten tritt nie auf“ bis 4 „Verhalten tritt häufig auf“ benutzt.

Aufgrund des unterschiedlichen Kontexts wurde das Konzept der *Belastungen durch Verhaltensauffälligkeiten des Kindes* bei Eltern und Lehrpersonen nicht mit den gleichen Items operationalisiert. Die Items referenzieren auf die unterschiedlichen Kontexte (Schule, Familie) mit vergleichbaren Inhalten. Bei den Lehrpersonen wurde das Konzept mit vier Items operationalisiert (Beispielitem: Das wütende, aufbrausende oder schädigende Verhalten des Kindes im Unterricht macht mir schwer zu schaffen. α = 0,93, *M* = 3,1, *SD* = 1,3, *N* = 152); Die Items stammen von einer erprobten Skala von Benini et al. (2017) und wurden adaptiert. Bei den Eltern enthielt die Skala ebenfalls vier Items (EBI, Beispielitem: Ich habe bisweilen das Gefühl, dass mich mein Kind pausenlos in Anspruch nimmt, α = 0,81, *M* = 3,5, *SD* = 1,1, *N* = 142; Tröster 2010). Beide Belastungskonzepte wurden auf einer Skala von 1 „trifft überhaupt nicht zu“ bis 6 „trifft voll und ganz zu“ eingeschätzt.

### Auswertungsstrategie

Für die Überprüfung der Übereinstimmung von Lehrpersonen- und Elterneinschätzungen wurden wegen leichter Verletzung der Normalitätsannahme Wilcoxon-Tests mit SPSS Version 27 und das für diesen Test übliche Effektstärkemaß *r* berechnet. Nach Cohen (1992) entspricht ein Wert von 0,1 einem schwachen Effekt, 0,25 einem mittleren Effekt und 0,4 einem großen Effekt. Da bei manchen Kindern Daten von mehr als einer Lehrperson vorlagen, wurde der Mittelwert der Lehrpersoneneinschätzungen pro Kind verwendet. Danach wurden Spearman-Korrelationen zwischen allen involvierten Konzepten berechnet. Zur anschließenden Hypothesenprüfung kamen Strukturgleichungsmodelle (SEM) mit Mplus 8,2 zur Anwendung (Muthén und Muthén 1998–2017). Die Messmodelle wurden so definiert, dass jeweils eine latente Variable mehrere Indikatoren erklärte. Aufgrund von inhaltlicher Ähnlichkeit wurden folgende Fehlerkorrelationen zwischen zwei Indikatoren zugelassen: Bei der proaktiven Aggression wurden aus Lehrpersonen- und Elternperspektive die Residuale zweier Items korreliert. Zudem wurden bei den Belastungen aus Elternperspektive die Residuale von zwei Items korreliert. Um die Mehrebenenstruktur der Lehrpersonendaten zu kontrollieren, wurde die Funktion in Mplus 8.2 type=complex verwendet. Bei den Elterndaten wurde die Mehrebenenstruktur nicht kontrolliert. Weil die Einschätzung der reaktiven und proaktiven Aggression bei den Lehrpersonen von der Normalverteilung leicht abwich, wurde der robuste MLR-Schätzer verwendet (Muthén und Satorra 1995). Fehlende Werte wurden mit dem Little’s MCAR-Test überprüft und waren sowohl bei Lehrpersonen (χ^2^[41] = 25.1, *p* = 0,976) als auch Eltern (χ^2^[183] = 200,5, *p* = 0,178) zufällig verteilt. Daher wurden sie mit der Maximum Likelihood Methode, implementiert in MPlus 8.2, bearbeitet (Graham 2009).

Die Modellgüte wurde aufgrund des χ^2^-Wertes, des Quotienten *χ*^2^/df, des Comparative Fit Index (CFI) und des Root Mean Square Error of Approximation (RMSEA) geschätzt (Hu und Bentler 1999). Ein nicht signifikanter χ^2^-Wert indiziert eine gute Passung zwischen dem Modell und den Daten. Allerdings tendieren χ^2^-Werte bei größeren Stichproben dazu, signifikant zu werden. Die Messmodelle sowie die Strukturgleichungsmodelle wurden über folgende Gütekriterien nach Schermelleh-Engel et al. (2003) beurteilt: Comparative Fit Index (CFI ≥ 0,95), Root Mean Square Errors of Approximation (RMSEA ≤ 0,08), Standardized root mean square residual (SRMR ≤ 0,10) und Quotient von χ^2^ und Freiheitsgraden (1 ≤ χ^2^/df ≤ 3) gelten als akzeptabel. Marsh et al. (2004) betonen, dass die Modellgüte aufgrund des Gesamtbildes verschiedener Indizes beurteilt werden muss, weshalb ein Modell gegebenenfalls akzeptiert werden kann, auch wenn ein einzelner Fit-Index außerhalb dieser Grenze liegt. Weil gerichtete Hypothesen formuliert wurden, wird das Signifikanzniveau für gerichtete Hypothesen genannt.

## Ergebnisse

Zur Überprüfung der Hypothese 1b (Lehrpersonen schätzen reaktive und proaktive Aggression der Kinder höher ein als Eltern) wurden Medianvergleiche berechnet. In Tab. 1 sind die Einschätzungen von Lehrpersonen und Eltern dargestellt. Die Ergebnisse zeigen, dass gemäß Wilcoxon Tests die reaktive und proaktive Aggression der Kinder von Lehrpersonen signifikant höher eingeschätzt wurden als von Eltern (reaktive Aggression: *z* = 7,5, *p* < 0,001, *N* = 144,* r* = 0,62; proaktive Aggression: *z* = 8,1, *p* < 0,001, *N* = 143, *r* = 0,67). Gleichzeitig wurden von den Eltern die geringe Emotionsregulation tiefer und das kooperative Verhalten höher eingeschätzt als von Lehrpersonen (geringe Emotionsregulation: *z* = 3,5, *p* < 0,001, *N* = 146, *r* = 0,29; kooperatives Verhalten: *z* = −6,2, *p* < 0,001, *N* = 145, *r* = 0,51) (H1b bestätigt).

**Table 1:** 

	Lehrpersonen		Eltern		
	*N*	*Mdn*	*N*	*Mdn*	Sig
Reaktive Aggression	152	4,33	146	3,00	***
Proaktive Aggression	152	2,86	145	1,67	***
Geringe Emotionsregulation	153	3,50	147	3,25	***
Kooperatives Verhalten	152	3,00	147	3,40	***

*N* Anzahl Antwortende, *Mdn* Median, *Sig.* Signifikanzniveau

*p < 0,05, **p < 0,01, *** *p* < 0,001, zweiseitige Signifikanzprüfung

In Tab. 2 sind die Spearman-Korrelationen zwischen allen involvierten Konzepten aus Lehrpersonen- und Elternperspektive ersichtlich. Die meisten Korrelationen waren substanziell und signifikant.

**Table 2:** 

	2	3	4	5	6	7	8	9	10
1 E: Reaktive Aggression	0,52***	0,56***	−0,42***	0,54***	0,30***	0,34***	0,31***	−0,10 ns	0,22**
2 E: Proaktive Aggression	1	0,35***	−0,29***	0,36***	0,19*	0,21*	0,22**	0,02 ns	0,12 ns
3 E: Geringe Emotionsregulation	–	1	−0,35***	0,54***	0,11 ns	0,22**	0,24**	−0,10 ns	0,15 ns
4 E: Kooperatives Verhalten	–	–	1	−0,31***	−0,17*	−0,18*	−0,15 ns	−0,15 ns	0,08 ns
5 E: Belastung	–	–	–	1	0,15 ns	0,27***	0,20*	−0,21*	0,31**
6 L: Reaktive Aggression	–	–	–	–	1	0,54***	0,57***	−0,37***	0,33***
7 L: Proaktive Aggression	–	–	–	–	–	1	0,30***	−0,25**	0,30***
8 L: Geringe Emotionsregulation	–	–	–	–	–	–	1	−0,42***	0,53***
9 L: Kooperative Verhalten	–	–	–	–	–	–	–	1	−0,28***
10 L: Belastung	–	–	–	–	–	–	–	–	1

*E* Elternperspektive, *L* Lehrpersonenperspektive, *N* = 142–152

*p < 0,05, **p < 0,01, *** *p* < 0,001, ns: *p* > 0,05

**Figure 1:**
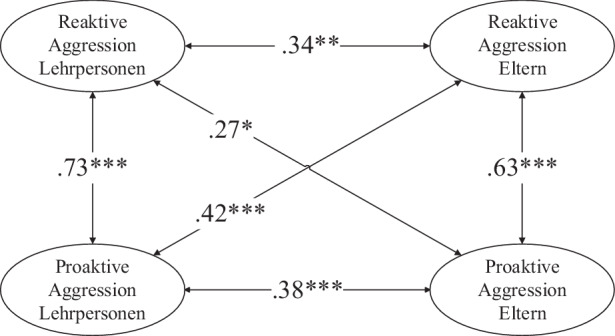

Zur Prüfung der Hypothese 1a, wonach die Einschätzungen der reaktiven und proaktiven Aggression von Eltern und Lehrpersonen miteinander korrelierten, wurde ein Strukturgleichungsmodell berechnet (χ^2^ = 76,2, df = 46, *p* < 0,05, χ^2^/df = 1,7, CFI = 0,95, RMSEA = 0,07, SRMR = 0,05, *N* = 154, Abb. 1, Darstellung ohne Messmodelle). Die Einschätzungen der reaktiven und proaktiven Aggression stimmten aus Eltern- und Lehrpersonenperspektive überein (0,27 < φ < 0,42). Die Korrelationen zwischen der reaktiven und der proaktiven Aggression waren aus Perspektive der Eltern (φ = 0,63) und der Lehrpersonen (φ = 0,73) signifikant (H1a bestätigt).

**Figure 2:**
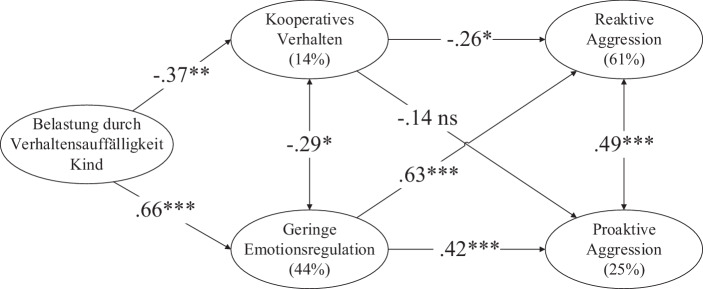

Ein weiteres Strukturgleichungsmodell wurde zur Überprüfung der Hypothesen 2 bis 4 berechnet (χ^2^ = 184,6, df = 142, *p* < 0,01, χ^2^/df = 1,3, CFI = 0,95, RMSEA = 0,05, SRMR = 0,06, *N* = 147). In Hypothese 2 wird postuliert, dass Emotionsregulation und kooperatives Verhalten von Kindern negativ mit proaktiver und reaktiver Aggression korrelieren. Hypothese 3 nimmt an, dass hohe Belastungen der Lehrpersonen bzw. der Eltern einen negativen Effekt auf kooperatives Verhalten und die Emotionsregulation haben. Hypothese 4 sagt aus, dass die Effekte der Belastungen von Lehrpersonen bzw. Eltern auf die reaktive und proaktive Aggression durch kooperatives Verhalten und Emotionsregulation mediiert werden. Abb. 2 (Darstellung ohne Messmodelle) zeigt aus der Elternperspektive signifikante Zusammenhänge zwischen geringer Emotionsregulation und reaktiver und proaktiver Aggression sowie zwischen kooperativem Verhalten und reaktiver Aggression (H2 bestätigt). Ebenfalls fanden sich signifikante Zusammenhänge zwischen den Belastungen und dem kooperativen Verhalten (−) bzw. der geringen Emotionsregulation (H3 bestätigt). Die totalen indirekten Effekte von den Belastungen auf die reaktive Aggression (β = 0,51, *p* < 0,001) und proaktive Aggression (β = 0,33, *p* < 0,001; H4 bestätigt) waren signifikant.

Zur Prüfung der Robustheit wurde das Modell unter der Annahme neu gerechnet, dass das Geschlecht einen direkten Effekt auf alle latenten Variablen aufweist. Der Effekt des Geschlechts auf die proaktive Aggression war signifikant (*p* < 0,05), auf alle anderen latenten Variablen war er nicht signifikant. Die Signifikanz der Pfade zwischen den latenten Variablen blieb nach Einbezug der Kontrollvariable Geschlecht gleich. Das Geschlecht beeinflusst offenbar die berichteten Effekte nicht.

**Figure 3:**
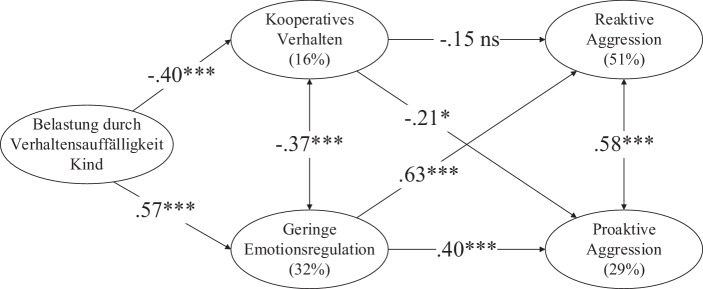

Das Modell aus der Lehrpersonenperspektive (χ^2^ = 246,7, df = 143, *p* < 0,001, χ^2^/df = 1,7, CFI = 0,93, RMSEA = 0,07, SRMR = 0,07, *N* = 153, Abb. 3) zeigte analoge Ergebnisse zur Abb. 2 mit zwei Ausnahmen: Der Effekt vom kooperativen Verhalten auf die reaktive Aggression war nicht signifikant, während der Effekt vom kooperativen Verhalten auf die proaktive Aggression signifikant war (H5 teilweise bestätigt). Die totalen indirekten Effekte von der Belastung auf die reaktive Aggression (β = 0,41, *p* < 0,001) und auf die proaktive Aggression (β = 0,31, *p* < 0,001) waren signifikant.

Ein Robustheitscheck mit dem Geschlecht wurde auch für das Modell aus Lehrpersonenperspektive durchgeführt. Die Ergebnisse waren gleich, d. h. das Geschlecht hatte einen signifikanten Effekt auf die proaktive Aggression (*p* < 0,05), nicht aber auf die anderen latenten Variablen. Der Einbezug des Geschlechts veränderte das Signifikanzniveau der Effekte zwischen den latenten Variablen sowie die dargestellten Effekte zwischen den latenten Variablen nicht.

## Diskussion

Die Ergebnisse der Strukturgleichungsmodelle zeigen, dass Belastungen der Eltern bzw. Lehrpersonen aufgrund von Verhaltensauffälligkeiten der Kinder signifikant mit der Emotionsregulation und dem kooperativen Verhalten von Kindern im Kindergarten sowie in der Primarstufe zusammenhängen. Sie haben einen indirekten Effekt auf die reaktive und proaktive Aggression. Die Ergebnisse aus der Perspektive der Lehrpersonen lassen sich mit den Ergebnissen aus der Perspektive der Eltern weitgehend replizieren. Die Einschätzungen der reaktiven und proaktiven Aggression korrelierten zwischen der Eltern- und der Lehrpersonenperspektive, jedoch schätzten Lehrpersonen die reaktive und proaktive Aggression der Kinder signifikant höher ein als die Eltern. Dies könnte darauf hinweisen, dass Lehrpersonen auf die reaktive und proaktive Aggression der Kinder im Unterricht sensibler reagieren als Eltern im familiären Umfeld (Abikoff et al. 1993; Hintermaier et al. 2019). Die Ergebnisse der vorliegenden Untersuchung, die auf einer Stichprobe von Kindern mit Verhaltensauffälligkeiten basieren, stimmen mit Annahmen des Modells der Informationsverarbeitung von Crick und Dodge (1994) überein.

Während Verhaltensauffälligkeiten als Ursprung von Belastungen der Lehrpersonen und Eltern immer wieder analysiert wurden (Amstad und Müller 2020), postuliert die vorliegende Analyse einen Kreisprozess (Bottiani et al. 2019; Crick und Dodge 1994), wonach Verhaltensauffälligkeiten von Kindern zu Belastungen von Lehrpersonen bzw. Eltern führen, die wiederum das Risiko von proaktiver und reaktiver Aggression erhöhen (Siekkinen et al. 2013). Obwohl dieser Kreisprozess empirisch nicht überprüft werden konnte, zeigen die Ergebnisse erstmals, dass der Effekt der Belastungen von Lehrpersonen bzw. Eltern auf die reaktive und proaktive Aggression durch die Emotionsregulation und das kooperative Verhalten aus beiden Perspektiven mediiert wird. Durch die Förderung der Emotionsregulation und des kooperativen Verhaltens ergeben sich daher Möglichkeiten, den Teufelskreis von Verhaltensauffälligkeiten – Verstärkung der Belastungen – Verstärkung der Verhaltensauffälligkeiten zu durchbrechen.

Hypothesenwidrig hat aus Elternperspektive das kooperative Verhalten keinen Effekt auf die proaktive Aggression der Kinder. Eltern schätzen das kooperative Verhalten sowie die reaktive und proaktive Aggression positiver als Lehrpersonen ein. Auch Hintermaier et al. (2019) zeigten, dass Fachlehrpersonen die sozio-emotionale Entwicklung, insbesondere die proaktive Aggression, von Kindern mit Beeinträchtigungen tiefer einschätzten als Mütter. Vigil-Colet et al. (2012) zeigten, dass die Einschätzung von aggressivem Verhalten sozialer Erwünschtheit unterliegt. Die Korrelation vom kooperativen Verhalten auf die proaktive Aggression aus Elternperspektive wurde in den vorliegenden Ergebnissen nicht signifikant. Möglicherweise unterliegen die Elterneinschätzungen bezüglich proaktiver Aggression stärker der sozialen Erwünschtheit als Lehrpersoneneinschätzungen, weil Eltern den Einsatz von proaktiver Aggression als normwidriger interpretieren als die reaktive Aggression (Raine et al. 2006).

Aus Lehrpersonenperspektive hängt das kooperative Verhalten hypothesenwidrig nicht mit der reaktiven Aggression zusammen. Die Lehrpersonen schätzen die reaktive Aggression der Kinder im Vergleich zu den Eltern höher ein. Möglicherweise führt die hohe Sensibilität der Lehrpersonen gegenüber reaktiver Aggression von Kindern zu verzerrten Einschätzungen (Abikoff et al. 1993), die sich in einer fehlenden Korrelation zwischen dem kooperativen Verhalten und der reaktiven Aggression widerspiegeln.

Die Interpretation der präsentierten Ergebnisse unterliegt mehreren Limitationen. Das verwendete Querschnittdesign ermöglicht lediglich Zusammenhangsaussagen und keine Kausalaussagen. Die postulierten Richtungen der Effekte sind theoretische Annahmen, deren Kausalitätsrichtung empirisch nicht überprüft werden konnte. Die Fremdeinschätzungen der Eltern und Lehrpersonen weichen von der Selbstwahrnehmung des Kindes, die nicht untersucht wurde, möglicherweise ab. Die Validität der Einschätzungen konnte aber insofern bestätigt werden, dass sie bei Eltern und Lehrpersonen in hohem Maß übereinstimmen, obwohl sie sich auf zwei verschiedene Kontexte (Familie und Schule) beziehen. Nicht abschließend geklärt werden konnte die Frage, ob die unterschiedlichen Einschätzungen von Eltern und Lehrpersonen auf Verhaltensunterschieden der Kinder in Schule und Familie basieren, oder ob sie die unterschiedliche Sensibilität von Eltern und Lehrpersonen widerspiegeln. Da die Korrelationen trotz der Mittelwertsunterschiede signifikant sind, handelt es sich bei den Mittelwertunterschieden möglicherweise um Wahrnehmungseffekte (Youngstrom et al. 2000).

### Schlussfolgerungen für Forschung und Praxis

Studien, die ausschließlich Kinder mit Verhaltensauffälligkeiten in die Stichprobe einschließen, sind selten, obschon die Aussagekraft im Hinblick auf die Prävention und Intervention bei der Reduktion von Verhaltensauffälligkeiten höher sein dürfte. Zukünftige Forschung sollte daher vermehrt klinische Stichproben einbeziehen und die Kreisprozesse von Verhaltensauffälligkeiten, belasteten Bezugspersonen, Informationsverarbeitung, reaktiver und proaktiver Aggression längsschnittlich analysieren. Während Effekte von Verhalten der Kinder auf die Belastungen der Lehrpersonen und Eltern häufig untersucht wurden, wurden Effekte von Belastungen der Bezugspersonen auf das Verhalten der Schüler*innen selten bearbeitet (Jennings und Greenberg 2009; Siekkinen et al. 2013). Ebenso könnte sich die Weiterführung einer kontextvergleichenden Analyse in Schule und Familie in zukünftigen Studien lohnen, weil sie aufzeigt, wie unterstützende bzw. hemmende Prozesse in der Schule und in der Familie in Wechselwirkung miteinander stehen (Neuenschwander et al. 2005). Die Analyse dieser Wechselwirkungen zwischen Schule und Familie dürfte sich insbesondere beim Eintritt in den Kindergarten lohnen, wenn die Kinder mit neuen schulischen Erfahrungen und Normen (zum Beispiel langes, ruhiges Sitzen am Pult, Einhalten von Regeln u. a.) konfrontiert werden.

Für die Prävention von reaktiver und proaktiver Aggression bei Kindern mit belasteten Bezugspersonen verweisen die Ergebnisse auf die hohe Bedeutung der Emotionsregulation und des kooperativen Verhaltens. Die Förderung der Emotionsregulation und des kooperativen Verhaltens kann zur Reduktion von reaktiver und proaktiver Aggression in der Kindheit beitragen. Emotionsregulation und kooperatives Verhalten werden beispielsweise im FOSSA-Programm (Neuenschwander et al. 2022) oder im Programm Faustlos (Schick und Cierpka 2005), das auch auf dem Konzept der Informationsverarbeitung von Crick und Dodge (1994) basiert, gefördert. Eine frühe Förderung der Emotionsregulation und des kooperativen Verhaltens könnte aggressives Verhalten in der Schule und in der Familie reduzieren.
